# Answering the Call for Community Pharmacists to Improve Healthcare Delivery to Trans and Gender Diverse People: Guide for Designing, Implementing, and Evaluating an Online Education Program in Australia

**DOI:** 10.3390/pharmacy12010007

**Published:** 2023-12-31

**Authors:** Swapna Chaudhary, Robin A. Ray, Beverley D. Glass

**Affiliations:** College of Medicine and Dentistry, James Cook University, Townsville, QLD 4811, Australia; robin.ray@jcu.edu.au (R.A.R.); beverley.glass@jcu.edu.au (B.D.G.)

**Keywords:** pharmacy, pharmacists, transgender, gender diverse, education, trans and gender diverse health

## Abstract

Background: Trans and gender-diverse people visiting pharmacies may not always receive optimum care due to pharmacists’ lack of knowledge and confidence to provide such care. This situation prompts a need for training. Objectives: This paper aimed to describe a guide to the design, implementation, and evaluation of a training program on transgender healthcare for pharmacists in Australia. Methods: The Implementation Mapping Framework provided a foundation for the design, implementation, and evaluation of this training program. Through active involvement in the program development, trans and gender diverse people and pharmacists guided the program design, ensuring alignment with the cultural, social, and healthcare contexts. Results: The needs analysis highlighted the necessity for training for pharmacists to improve their cultural awareness and pharmacotherapeutic knowledge about transgender healthcare. Applying a novel Gender Inclusivity in Pharmacy Framework, online modules—(1) Transgender healthcare—language, terminology, and key healthcare issues, (2) Gender-affirming therapies, and (3) Case studies in transgender healthcare—were developed to enable the implementation of a training program. Conclusion: The Implementation Mapping Framework and the Gender Inclusivity in Pharmacy Framework proved effective tools for providing an education program for pharmacists.

## 1. Introduction

Greater recognition and representation of trans and gender diverse people has resulted in an increased demand for healthcare services for people in these marginalised communities [1]. Trans and gender diverse describes people whose gender identity differs from their sex assigned at birth and encompasses those who may identify as transgender, non-binary, sistergirl, brotherboy or other gender identities outside the traditional gender binary [2]. Although trans and gender diverse people visit pharmacies to obtain pharmaceutical care and advice [3,4,5], many pharmacists have not received training and do not feel confident and comfortable when providing such care and advice to trans and gender diverse people [5,6,7] 

Research has highlighted the existence of health disparities among trans and gender diverse people [8]. Pharmacists have a role to play in addressing these healthcare disparities experienced by people belonging to the trans and gender diverse communities [9,10,11]. However, the provision of optimal care to trans and gender diverse people by pharmacists may be impacted by their lack of cultural awareness and pharmacotherapeutic knowledge specific to this population [3,4,7,10,11]. Trans and gender diverse people have shared their negative experiences with pharmacists and pharmacy staff when seeking pharmaceutical care from community and hospital pharmacy settings [3,4,5]. Such encounters perpetuate the stigmatization and marginalization of an already vulnerable community and discourage trans and gender diverse people from accessing the available healthcare services that could potentially address their unique health needs [4,10,12]. Pharmacists need to acquire the necessary sensitivity and competence to deliver inclusive and affirming care [10,11,13].

The Pharmaceutical Society of Australia’s (PSA) Pharmacy Practice Standards and Code of Ethics expect pharmacists to deliver respectful, inviting, non-discriminatory, and evidence-based care to all clients [14,15]. Moreover, the “Equality Position Statement” from the PSA emphasizes the importance of equitable access to inclusive and discrimination-free healthcare for lesbian, gay, bisexual, transgender, queer, intersex and asexual+ (LGBTQIA+) people, advocating for integrating LGBTQIA+ health education into all pharmacy programs in Australia [16]. Additionally, pharmacists are encouraged to use respectful and inclusive language while providing appropriate care to people who are part of LGBTQIA+ communities [16]. However, currently, there is no training program available in trans and gender diverse healthcare for pharmacists in Australia. 

Globally, trans and gender diverse healthcare educational interventions ranging from one-hour to five-hour sessions for pharmacists and pharmacy students have successfully improved knowledge and skills in providing care to trans and gender diverse people [17,18,19,20,21]. A study from Puerto Rico has been shown to improve pharmacists’ knowledge about trans and gender diverse care and pharmacotherapeutic options for gender affirmation [21]. An American study reported using a flipped classroom model where students completed pre-class activities such as reading and watching a pre-recorded lecture and then in class they participated in other activities such as role plays, a game-style learning activity, and engaged with conversation with trans and gender diverse people [17]. A few other studies included didactic lectures [20,22], discussion with trans and gender diverse panels [18,20], videos [18], and game-style activities [18]. All these studies improved pharmacy students’ knowledge and confidence in providing care for trans and gender diverse people [17,18,20,22]. While international interventions can offer insights, it is important to meet the needs of both Australian trans and gender diverse people and pharmacists, with a program developed within the Australian context, providing education focussed on trans and gender diverse healthcare to enhance their capability to serve these populations.

Previous scoping review and mixed method research among pharmacists and trans and gender diverse people identified the need for this training [3,6,7,9]. This article aims to describe a guide to the design and implementation along with an evaluation plan of a training program on trans and gender diverse healthcare for pharmacists in Australia using an Implementation Mapping Framework [23]. This research required a framework that provided a step-by-step but comprehensive and participatory approach to design, implement, and evaluate the training program. The Intervention Mapping Framework involves active participation of stakeholders and provides a systematic and comprehensive approach to identify, plan, and execute the necessary steps for successful program design and implementation. This framework was chosen as it aligned well with our research objectives. Other frameworks [24,25] were considered; however, they could be only partially applied to the design of this study.

## 2. Materials and Methods

The Implementation Mapping Framework [23] provided a foundation for designing, implementing, and evaluating a training program on trans and gender diverse healthcare for pharmacists. This program is intended to meet the diverse needs of trans and gender diverse people and equip pharmacists with the knowledge and communication skills necessary to provide inclusive and affirming care. The Intervention Mapping Framework’s structured, collaborative, and inclusive approach increases the likelihood of the program being appropriate, comprehensive, and evidence-based [23].

**Figure 1:**
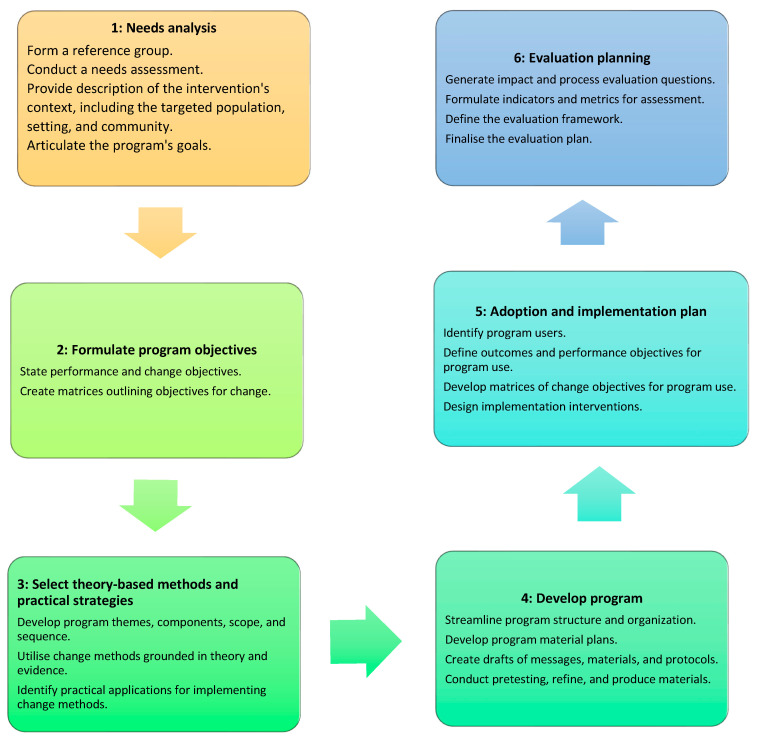
Six Steps of Intervention Mapping Framework [22].

This framework provides six sequential steps to guide the design of educational and health promotion interventions (Figure 1): (1) Needs analysis, (2) Formulate program objectives, (3) Select theory-based methods and practical strategies, (4) Develop intervention, (5) Adopt and implement program, and (6) Plan for evaluation [23]. Through active involvement in the program development process, trans and gender diverse people and pharmacists guided the program design, ensuring alignment with the cultural, social, and healthcare contexts.

Step 1: Needs analysis.

A needs analysis has been performed to explore the role of pharmacists, including their attitudes and practices in trans and gender diverse care, and the experiences and expectations of trans and gender diverse people receiving pharmaceutical care, to identify pharmacists’ training needs in trans and gender diverse care [3,6,7,9]. A literature search and data from stakeholder interviews and surveys identified the behavioural and environmental changes needed and the reasons for each change to pharmacy practice to enhance the provision of appropriate care to trans and gender diverse people [3,6,7,9]. This information was then used to guide the program’s design, including the development of the program goals and performance objectives. The training program was designed in consultation with a reference group of three trans and gender diverse community members, three pharmacists, and three academics. The overall goal of the program was to improve cultural awareness and pharmacotherapeutic knowledge of pharmacists to provide respectful and gender-affirming care for trans and gender diverse people.

Step 2: Formulate program objectives.

Based on the program goals from the previous step, the performance objectives (Table 1) were developed, detailing the possibilities for practice transformation after implementing the intervention [23]. The determinants of trans and gender diverse healthcare in a pharmacy requiring change to provide appropriate healthcare to trans and gender diverse people in pharmacy were utilised to inform the performance objectives. The performance objectives included the personal determinants (knowledge, awareness, and attitudes) and non-behavioural environmental factors (physical pharmacy environment) that needed to change to attain the overarching program goal.

Step 3: Select theory-based methods and practical strategies.

**Figure 2:**
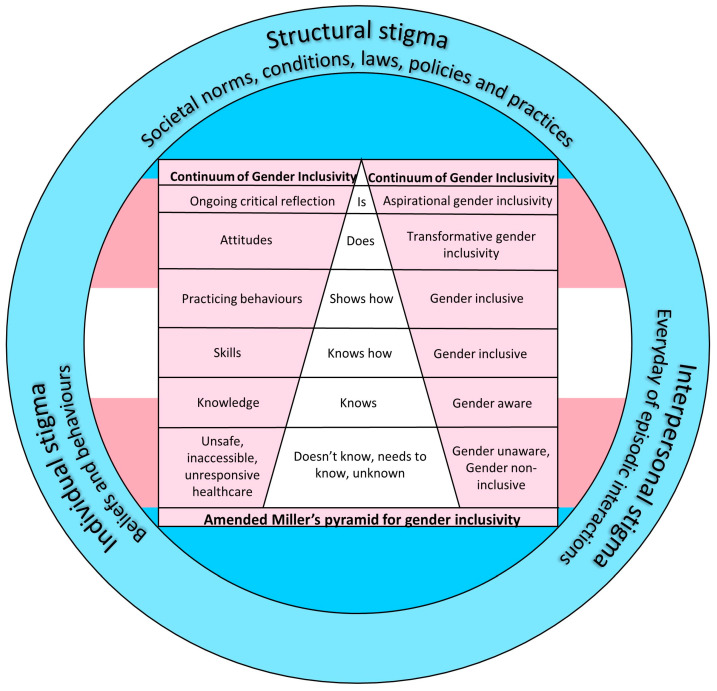
Gender Inclusivity in Pharmacy Framework.

Educational and trans and gender diverse care theories were chosen to accomplish the program objectives defined in Step 2. These theories informed the design of a novel Gender Inclusivity in Pharmacy Framework (Figure 2) for attaining the program objectives. This framework guided the program’s development (Step 4) and evaluation (Step 6). The identified determinants requiring change were mapped against performance objectives, with theory-based methods and practical strategies utilised to develop and implement the program.

Step 4: Develop intervention.

The training materials were developed in consultation with the reference group members by applying the goals, objectives, and learning theory produced in previous steps. A training platform was chosen to build the program.

Step 5: Adoption and implementation plan.

Planning for implementation of the program began at the needs analysis step. Pharmacists were recognised as the target groups for this program, and strategies to recruit these participants and disseminate information about the program were also identified. 

Step 6: Evaluation planning.

Strategies to evaluate the effectiveness and outcomes of the program were designed to capture the impact of the training on behavioural and environmental determinants of pharmaceutical trans and gender diverse care. An amended Miller’s pyramid [26] for competency assessment was utilised to measure the changes in knowledge, skills, behaviours, and attitudes of the participants completing the training program. 

## 3. Results

Step 1: Needs analysis.

The scoping review [9] highlighted the role of pharmacists in trans and gender diverse care, including educating and counselling patients, managing gender-affirming therapy, advocating for patients, and providing preventative care. They recognised their role in providing culturally sensitive care and a welcoming environment for trans and gender diverse people [9]. Although they were aware of their significant role in trans and gender diverse care, they expressed a lack of confidence in their knowledge about providing care to this population group [9]. As a result of this review, we know that pharmacists need specialised education in trans and gender diverse care to ensure that both are confident and comfortable in providing care to trans and gender diverse people.

The interview-based needs analysis with trans and gender diverse people who were recipients of pharmacy care revealed two predominant themes: (1) Difficulties encountered in seeking care from pharmacies, and (2) Maximizing the quality of interactions between trans and gender diverse people and pharmacists [3]. According to the findings, trans and gender diverse people expressed concerns about experiencing anxiety while seeking care, facing constraints within the healthcare system, privacy and confidentiality issues at the pharmacy, and encountering challenges related to their gender identity. These factors were identified as significant reasons leading trans and gender diverse people to either avoid or limit their interactions with pharmacists and pharmacy staff, highlighting the multifaceted barriers that contribute to the complexities of healthcare access for this demographic [3]. 

Furthermore, pharmacists’ attitudes, practices, and training needs in providing care for trans and gender diverse people in Australia were explored through pharmacists’ interviews and a national survey [6,7]. Pharmacists displayed a positive attitude towards providing care to trans and gender diverse people and acknowledged the importance of their role in trans and gender diverse care [6,7]. However, they considered their limited knowledge of trans and gender diverse healthcare as a critical barrier to providing quality care to trans and gender diverse people [6,7]. Given the inadequate or limited coverage of trans and gender diverse care in pharmacy curricula and CPE activities, most pharmacists suggested the addition of education about trans and gender diverse healthcare, and specifically cultural and pharmacotherapeutic education in both pharmacy curricula and CPE activities [6,7].

Trans and gender diverse people and pharmacists expressed the need for education to provide care to trans and gender diverse people which informed the program goal: to improve awareness and knowledge about trans and gender diverse healthcare to pharmacists by implementing a program that would enhance their communication skills and confidence in their knowledge of gender-affirming therapies. Moreover, incorporating the lived experiences of trans and gender diverse people in a program that could be delivered online was essential to enhancing the attitudes and practices of pharmacists about providing care to trans and gender diverse people.

Step 2: Formulate program objectives.

Considering pharmacists’ role in trans and gender diverse care and the PSA’s Professional Practice Standards for pharmacists in Australia [14], performance objectives for pharmacists participating in this program were developed (Table 1). These performance objectives were designed to improve the awareness and knowledge of pharmacists in trans and gender diverse healthcare through comprehending culturally sensitive language, as well as healthcare issues of trans and gender diverse people and gender-affirming therapies, thus aligning with the program goal. An online delivery mode was selected for the modules, and practical strategies were chosen considering the program objectives [12,16,26,27]. The practical strategies comprised written information and case studies supplemented by learning videos involving trans and gender diverse people and pharmacists.

Step 3: Theory-based methods and practical strategies.

In this stage, the process entailed selecting methods and practical strategies grounded in established theories (Table 1) for inclusion in the design of the training program. Based on the Gender Equity Framework for Transgender Populations [23], a Modified Social-Ecological Model of Transgender Stigma and Stigma Intervention [12], and an Amended Miller’s pyramid [26], we developed a conceptual framework—Gender Inclusivity in Pharmacy as a novel way to guide the design of this training program (Figure 2). This framework was adapted from the study on Indigenous cultural safety by Brumpton et al. [28].

This novel Gender Inclusivity in Pharmacy Framework was utilised to design the components of the program that will promote the acquisition of knowledge, skills, and attitudes necessary to address individual, interpersonal, and structural stigma and enhance gender inclusivity for trans and gender diverse people in pharmacy. 

The outer blue wheel indicates that promoting gender inclusivity for trans and gender diverse people in pharmacy can be achieved through targeted education to reduce three types of stigmas—individual, interpersonal, and structural [12]. Addressing individual stigma involves providing comprehensive training for pharmacists about the diverse spectrum of gender identities and the importance of respectful language and interactions [12,13]. Interpersonal stigma reduction can be achieved by fostering a supportive and understanding environment where pharmacists actively engage in respectful and empathetic interactions with trans and gender diverse people, focusing on their specific healthcare needs [12,16]. To combat structural stigma, educational initiatives can highlight the significance of creating policies and procedures that respect trans and gender diverse rights, ensuring the availability of gender-affirming medications, and advocating for inclusive laws that protect trans and gender diverse people from discrimination [12,13,16]. By addressing these three dimensions of stigma through education, pharmacies can become more welcoming and inclusive spaces for trans and gender diverse people seeking pharmaceutical care.

The amended Miller’s pyramid [24] provided a structured approach to developing and evaluating the clinical competence of training participants in trans and gender diverse care across four levels: knowledge, skills, behaviours, and attitudes. The program was designed so that the gender inclusivity level for all participants would improve from ‘Gender unaware’ to being ‘Gender inclusive’ [29]. Starting at the foundational level of knowledge, the program focused on providing pharmacists with a comprehensive understanding of trans and gender diverse health, including terminology, hormone therapies, and potential drug interactions so that they ‘know’ about trans and gender diverse care and become ‘Gender aware’ [26,29]. Moving up the pyramid, the program addressed the development of skills, such as effective communication with trans and gender diverse people and cultural competence, so that they become ‘Gender inclusive’ [26,29]. The next level involves translating knowledge and skills into behaviours, where the program emphasizes the application of learned concepts in real-world scenarios in the form of case studies so that the learners can see how to be ‘Gender inclusive’ [26,29]. It was anticipated that on completion of this program, pharmacist participants would undergo a significant transformation in their approach to providing care for trans and gender diverse people. This transformation, referred to as ‘Transformational Gender Inclusivity,’ involves substantial changes in their attitudes and practices. Furthermore, participants can aspire to achieve ‘Aspirational Gender Inclusivity’ by consistently and critically assessing their attitudes, behaviours, knowledge, and skills in delivering care for trans and gender diverse people [26,29].

Principles of adult learning theory [27] were applied to develop the components of the training program. Knowles describes how adult learners can create new learning based on their previous experiences and understandings [27]. They are keen to learn new material when they recognise its relevance to their lives [27]. This program design considered Knowles six assumptions [27] about adult learners (see Appendix A). Acknowledging the self-directed nature of adult learners while emphasizing the importance of autonomy in completing learning modules, this training program was designed to be completed on their own time.

Step 4: Program development.

Based on the previous three steps and consultation with the reference group, the principal investigator developed an online training program. The principal investigator has undertaken training [30,31,32] in trans and gender diverse healthcare and has experience providing care to trans and gender diverse people in pharmacy. Co-authors, who were experienced educators, reviewed the training material before launching the program. The online program consisted of the following three modules:

1. Transgender Healthcare—Language, terminology, and key healthcare issues.
2. Gender-affirming therapies.
3. Case Studies in trans and gender diverse healthcare.

The comprehensive training program equipped participants with a wealth of resources, encompassing reading materials, supplementary links, and pre-recorded videos. These videos showcased people within the trans and gender diverse community sharing insights into their personal pharmacy journeys and articulating their expectations concerning pharmaceutical care. Furthermore, the videos illustrated both inappropriate and appropriate interactions with trans and gender diverse individuals within community pharmacy settings. Specific scenarios, such as counselling on over-the-counter medicines and hormonal medications for gender affirmation, were addressed. Notably, these instructive videos were a result of collaborative efforts involving local trans and gender diverse volunteers and experienced community pharmacists.

Step 5: Adoption and implementation.

The final training program’s estimated time to completion would be eight hours, including the time taken to complete the pre-test and post-test evaluation surveys. The program modules will be uploaded to the organizational site called ‘Pharmacists in Transgender Healthcare’ as a component of the JCU Blackboard educational platform. This online platform will provide participants with easy navigation throughout the program material. All 126 pharmacists who expressed their interest in this program [6] will be enrolled. The pharmacist participants will have ten weeks to complete this self-paced program. 

Step 6: Evaluation plan.

Online pre-test and post-test surveys will be developed for administration using the Qualtrics platform [33] to assess the impact of this training program on participants’ awareness and knowledge of trans and gender diverse healthcare. These survey links will be integrated into the program and shared on the organizational site. Pharmacists must complete the pre-test survey to access the training modules, during which they will generate a unique code for subsequent access to the post-test survey. This matching code facilitates the comparison of each participant’s pre-test and post-test responses. To obtain a course completion certificate, participants are required to complete the post-test survey. Upon completion, pharmacists will receive a confidential link to provide their contact details (full name and email address) and express their interest in a three-month post-training outcome evaluation interview. After the three-month post training period, the principal investigator will email the pharmacists interested in the post-evaluation interview to assess the outcome of training on the pharmacists’ practices.

## 4. Discussion

This is the first study to provide a guide to designing, implementing, and evaluating a training program in trans and gender diverse healthcare for pharmacists using the intervention mapping framework [23]. Utilising the intervention mapping framework established a strong foundation that enabled a systematic progression in developing the training program components and their subsequent evaluation [23]. This framework proved advantageous as it allowed for the incorporation of comprehensive input and diverse viewpoints from stakeholders, resulting in the design of a program that effectively addressed the identified issues and gaps in pharmacists’ trans and gender diverse health education. Their collective participation provided practical insights that were based on their experiential knowledge playing a crucial role in shaping the intervention. The stepwise description from development to evaluation may serve as a guide for developing future training programs in trans and gender diverse healthcare and other healthcare training programs.

While intervention mapping [23] offered a robust methodology for developing this training program, implementing all its steps proved to be time consuming. The challenge was to create a training program that could effectively integrate diverse perspectives and offer a tailored approach grounded in theory, evidence, and practical experience to address the day-to-day situations that may arise in pharmacy while caring for trans and gender diverse people. The Gender Inclusivity in Pharmacy Framework facilitated the integration of theories, evidence, and practical strategies to address individual, interpersonal, and structural stigma experienced by the trans and gender diverse population. Integration of Miller’s pyramid [24] into this framework provided a comprehensive and progressive approach to evaluating the participants’ competency in the training program. 

A notable strength of this study was the involvement of the key stakeholders—trans and gender diverse community members and pharmacists—who would benefit from this program. Key stakeholder engagement throughout the program enabled the development of program objectives. Additionally, the stakeholder input may have improved program adoption and potentially enhanced the impact of the program. Another strength is that the program was designed by the principal investigator, who possesses both knowledge and training in trans and gender diverse care and relevant practical experience. This expertise was important in ensuring that the developed program was comprehensive and responsive to the specific needs of trans and gender diverse people. However, it is important to acknowledge that not all university programs may have immediate access to faculty members with such specialised training [34]. In such cases, alternative strategies can be employed to bridge this gap. One option is to hire individuals with expertise in trans and gender diverse care as adjunct faculty or consultants who can contribute their knowledge and guidance in developing the program [34]. Another approach is to actively engage with local trans and gender diverse community members and experienced pharmacists to gain insights and perspectives that can inform the objectives and content of the program. This collaborative effort will ensure that the training program remains relevant, inclusive, and aligned with the best practices in trans and gender diverse care, even without internal trained faculty.

In the future, this training program can be more widely disseminated in collaboration with educational institutes, professional organizations, and trans and gender diverse organizations. Program promotion and uptake can potentially improve healthcare professionals’ attitudes and knowledge to improve the provision of services that meet the needs of this marginalised population, thereby reducing the individual, interpersonal, and societal stigma experienced by trans and gender diverse people. This program teaches universal principles for improving communication and creating an inclusive and welcoming environment for trans and gender diverse people, which can be applied to any organization or business, regardless of its healthcare or non-healthcare nature, to foster the inclusion of trans and gender diverse people. The program may be useful in the development of awareness campaigns to reduce societal stigma. Such campaigns could be promoted during special days for the trans and gender diverse population, such as Transgender Awareness Day, Wear It Purple Day, International Day Against Homophobia, Biphobia, and Transphobia (IDAHOBIT) Day and Trans Remembrance Day.

## 5. Conclusions

The Pharmaceutical Society of Australia’s (PSA) Pharmacy Practice Standards and Code of Ethics expectation of pharmacists to deliver respectful, inviting, non-discriminatory, and evidence-based care to all clients has not been met by all pharmacists. A knowledge and skills gap about trans and gender diverse healthcare provision by pharmacists highlighted during a needs analysis led to the design of an online training program to bridge this gap in knowledge and skills. The Implementation Mapping Framework provided a useful step-by-step approach to guide the design, implementation, and evaluation. A novel Gender Inclusivity in Pharmacy Framework also provided guidance for developing and evaluating the program components that may improve the pharmacists’ knowledge, skills, attitudes, and behaviours, ultimately fostering competence in providing care for trans and gender diverse people in pharmacy. This framework can be utilised to develop and evaluate future training programs in trans and gender diverse healthcare.

## Figures and Tables

**Table 1:** Performance objectives and educational methods.

Determinants	Module	Performance Objectives	Practical Strategy *
Awareness—Culture and Healthcare Needs	Transgender Healthcare—Language, Terminology, and Key Health Issues	To understand the language and terminology for appropriate communication with trans and gender diverse people. To describe how to create a welcoming and inclusive pharmacy environment for trans and gender diverse people. To identify key health issues faced by trans and gender diverse populations. To understand the role of pharmacists in trans and gender diverse healthcare.	Partnership with trans and gender diverse people and pharmacists. Videos with trans and gender diverse people to incorporate their lived experiences in the program. Pharmacists and trans and gender diverse people pharmacy interaction videos. Written information.
Knowledge	Gender Affirming Therapies	To discuss the common approaches for gender affirmation. To describe the role of hormonal and surgical therapies in gender affirmation. To identify potential drug interactions. To understand the effect of hormonal therapy on laboratory values.	Written information.
Skills	Case Studies Transgender Healthcare	To apply the learning from Modules 1 and 2 to address the problems and challenges faced in providing care to trans and gender diverse people, making decisions based on the evidence given.	Partnership with trans and gender diverse people and pharmacists. Case studies. Videos demonstrating appropriate counselling and interactions with trans and gender diverse people in pharmacy.

* Delivery Method—All modules were offered online and included relevant references and resources about trans and gender diverse health.

## Data Availability

Data are contained within the article.

## Appendix A. Application of Knowles Six Principles to the Design of the Program

1. **Adults are self-directed:** Adult learners favour taking responsibility for their learning [27]. Therefore, it was essential to design a learning program that respects their autonomy and provides an opportunity to complete the modules in the participant’s own time.
2. **Experience:** Adults use their experiences to shape their learning [27]. Pharmacists have extensive experience in providing care to various clients in the pharmacy. Drawing on these experiences provided a foundation for new learning about TGD healthcare and enhanced their understanding of new content by establishing links to their previous knowledge and experience.
3. **Readiness to learn**: Adult learners learn when they are ready to learn and when the learning benefits their personal or professional goals [27]. The earlier research indicated that most pharmacists desired to improve their practice of providing care to TGD people [6,7]. Capitalizing on readiness enabled pharmacists to learn from theory as well as practical strategies to improve their competence in TGD healthcare.
4. **Problem-centred orientation:** Adult learners want to see if the new learning can resolve their current problems [27]. This program design Including communication strategies, TGD healthcare knowledge, and day-to-day pharmacy practice scenarios enabled participants to immediately implement changes to their practice.
5. **Motivation to learn:** For adult learners, intrinsic motivators such as improved self-esteem, job satisfaction, confidence and personal growth may play vital role in motivating them to learn new things [27]. Participation in the program was voluntary relying on the participant’s internal motivation to participate and complete the program. The participants received a certificate on completing the program and could count the program hours (up to eight hours) towards their continuing professional education (CPE) points, providing additional external motivation to complete this program.
6. **The need to know**: Adults actively engage in training programs that they perceive relevant to their practice [27]. The needs analysis showed that the pharmacist participants in this program envisaged that learning new concepts and strategies in TGD care would be relevant to their current practice [6,7].
